# Mobility-Focused Physical Outcome Measures Over Telecommunication Technology (Zoom): Intra and Interrater Reliability Trial

**DOI:** 10.2196/38101

**Published:** 2022-08-22

**Authors:** Marie-Louise Bird, Felix Peel, Matt Schmidt, Natalie A Fini, Emily Ramage, Brodie M Sakakibara, Dawn B Simpson, Carey Mather, Dominique A Cadilhac, Kiran D K Ahuja, Heather Bridgman, Coralie English

**Affiliations:** 1 Department of Physical Therapy University of British Columbia Vancouver, BC Canada; 2 School of Health Sciences College of Health and Medicine University of Tasmania Launceston Australia; 3 School of Health Sciences College of Health and Medicine University of Tasmania Hobart Australia; 4 Physiotherapy Department University of Melbourne Melbourne Australia; 5 School of Health Sciences University of Newcastle Callaghan Australia; 6 Occupational Science and Occupational Therapy University of British Columbia Kelowna, BC Canada; 7 School of Health Sciences and Priority Research Centre for Stroke and Brain Injury University of Newcastle Callaghan Australia; 8 Translational Public Health and Evaluation Division, Stroke and Ageing Research, Department of Medicine School of Clinical Sciences at Monash Health Monash University Heidelberg Australia; 9 Public Health, Stroke Division Florey Institute of Neuroscience and Mental Health University of Melbourne Heidelberg Australia; 10 Heart and Stroke Research Program Hunter Medical Research Institute Newcastle Australia

**Keywords:** reliability, mobile health, telemedicine, telehealth, rehabilitation, mobility, consultation, physical function, assessment, Zoom

## Abstract

**Background:**

Rehabilitation provided via telehealth offers an alternative to currently limited in-person health care. Effective rehabilitation depends on accurate and relevant assessments that reliably measure changes in function over time. The reliability of a suite of relevant assessments to measure the impact of rehabilitation on physical function is unknown.

**Objective:**

We aimed to measure the intrarater reliability of mobility-focused physical outcome measures delivered via Zoom (a commonly used telecommunication platform) and interrater reliability, comparing Zoom with in-person measures.

**Methods:**

In this reliability trial, healthy volunteers were recruited to complete 7 mobility-focused outcome measures in view of a laptop, under instructions from a remotely based researcher who undertook the remote evaluations. An in-person researcher (providing the benchmark scores) concurrently recorded their scores. Interrater and intrarater reliability were assessed for Grip Strength, Functional Reach Test, 5-Time Sit to Stand, 3- and 4-Meter Walks and Timed Up and Go, using intraclass correlation coefficients (ICC) and Bland-Altman plots. These tests were chosen because they cover a wide array of physical mobility, strength, and balance constructs; require little to no assistance from a clinician; can be performed in the limits of a home environment; and are likely to be feasible over a telehealth delivery mode.

**Results:**

A total of 30 participants (mean age 36.2, SD 12.5 years; n=19, 63% male) completed all assessments. Interrater reliability was excellent for Grip Strength (ICC=0.99) and Functional Reach Test (ICC=0.99), good for 5-Time Sit to Stand (ICC=0.842) and 4-Meter Walk (ICC=0.76), moderate for Timed Up and Go (ICC=0.64), and poor for 3-Meter Walk (ICC=–0.46). Intrarater reliability, accessed by the remote researcher, was excellent for Grip Strength (ICC=0.91); good for Timed Up and Go, 3-Meter Walk, 4-Meter Walk, and Functional Reach (ICC=0.84-0.89); and moderate for 5-Time Sit to Stand (ICC=0.67). Although recorded simultaneously, the following time-based assessments were recorded as significantly longer via Zoom: 5-Time Sit to Stand (1.2 seconds), Timed Up and Go (1.0 seconds), and 3-Meter Walk (1.3 seconds).

**Conclusions:**

Untimed mobility-focused physical outcome measures have excellent interrater reliability between in-person and telehealth measurements. Timed outcome measures took approximately 1 second longer via Zoom, reducing the reliability of tests with a shorter duration. Small time differences favoring in-person attendance are of a similar magnitude to clinically important differences, indicating assessments undertaken using telecommunications technology (Zoom) cannot be compared directly with face-to-face delivery. This has implications for clinicians using blended (ie, some face-to-face and some via the internet) assessments. High intrarater reliability of mobility-focused physical outcome measures has been demonstrated in this study.

## Introduction

Globally, many people suffer from health conditions that require ongoing care from health professionals [1]. Telehealth can enable an effective and equitable service to help overcome current pandemic-induced and preexisting geographical and service-related barriers to accessing health care systems [2]. Telehealth is any health service that is being implemented or provided over telecommunication technologies [3], including assessment or service provision using audio, video, or app-based communication [4]. The provision of services via telehealth has rapidly increased over the last 2 years [5]; however, measurement of the effectiveness of such services in rehabilitation is hampered by the lack of research on the reliability of clinically relevant assessments, and in particular, mobility-focused physical outcome measures, recorded over telehealth technologies.

A handful of studies with small numbers of participants (less than 20) have included an element of mobility in their telehealth reliability measures; for example, Sit to Stand for patients with liver transplant [6], and Timed Up and Go for patients with knee arthroplasty [7], patients with Parkinson disease [8], and those with heart failure [9]. Validation of the reliability of a comprehensive suite of mobility-focused outcome measures delivered by telehealth is particularly relevant to rehabilitation services, where accurate assessment and tracking of changes in patient status, especially remotely, is crucial [10].

Given the likelihood and opportunity for ongoing growth of telehealth services, the reliability of mobility-focused physical outcome measures completed via telehealth technology is of interest and needs to be further explored, and the differences in these measures compared to face-to-face delivery need to be investigated. Simultaneous measurement of telehealth and conventional assessments has the advantage of ensuring that there is no potential for variance in the state of the patient [10]. In this study, we aimed to determine the reliability of several commonly used mobility-focused physical outcome measures when delivered via telehealth in a healthy population of individuals aged 18-60 years. The specific research questions addressed were the following:

- What is the interrater reliability of mobility-focused physical outcome measures assessed face-to-face and via Zoom, simultaneously?
- What is the level of agreement between mobility-focused physical outcome measures recorded face-to-face and via telehealth, simultaneously?
- What is the intrarater reliability of mobility-focused physical outcome measures recorded via telehealth?

## Methods

### Study Design

This was an observational measurement study designed to measure the reliability of mobility-focused physical outcome measures recorded using telecommunication technology. All testing was performed in a locked room to prevent disruption during data collection at the University of Tasmania, Launceston, Australia, between August and September 2021. The participant data collection area was a carpeted room, 10 meters long, with a standard-height chair (45 cm), a table, a laptop, and the equipment needed for each physical assessment: marker cones, handheld dynamometer, and measuring tape.

### Participants and Recruitment

Participants were recruited via posters placed around the campus and emails from the university administration that provided information and contact details for the research staff. Inclusion criteria were the following: individuals aged 18-60 years, willing and safe to participate, as measured by the Adult Preexercise Screening System tool. Exclusion criteria were any ongoing illness or mobility issues that would prevent the ability to safely perform physical measures. A total of 30 participants met the inclusion criteria and participant recruitment ceased when the sample size was met.

### Ethics Approval

The study protocol was explained to participants, and written informed consent was obtained prior to study entry. Ethical approval for the study was obtained from the University of Tasmania Human Research Ethics Committee (project ID 21690).

### Sampling

A suite of 7 commonly used clinical and research mobility-focused physical outcome measures were assessed. Measures and the number of trials completed in the assessment are shown in Table 1.

**Table 1:** Physical outcome measures assessed in the reliability study.

Measurement items	Number of trials
5-Time Sit-To-Stand Test [11]	2
3-Meter Walk Test [12]	2
4-Meter Walk Test [13]	2
Timed Up and Go Test [14]	2
Grip Strength Test [15]	3
Functional Reach Test [16]	3
Static Balance Test [12]	1

The 3-Meter Walk Test used a standing start, and the timing started when the telehealth researcher said “go,” as this comprises part of the Short Physical Performance Battery protocol [12]. In contrast, the walking speed for measuring the 4-Meter Walk timing started when the participant passed a marker on the floor, so it was not dependent on the reaction of the participant to start and then the researcher to see that start (to record usual walking speed more closely).

### Data Collection Process

Two researchers (MLB and FP) concurrently recorded the participant’s performance in each outcome measure. MLB is a physiotherapist with 20 years of clinical experience, and FP is a postgraduate researcher with 3 years of experience in measuring these clinical outcome measures. One researcher was present in the room with the participant, while a second researcher provided the assessment instructions and recorded measurements via a standard Zoom meeting, in another room. The participant’s laptop (brand Dell; latitude 7480, with a 35-cm screen) was set up and connected to the remote evaluator, via a call through Zoom. All assessments were completed in view of the laptop’s camera. Concurrently, the researcher in the room with the participant, who had limited interaction with the participant and did not provide any instructions, also recorded results of each physical assessment independently. Both researchers recorded results for each assessment on a paper-based form, which was later transcribed into a database; they also took field notes on the quality of assessments and so as to capture any potential issues with the technology. The measures were performed in a random order to account for any participant fatigue and to reduce any ordering bias. Randomization was undertaken via a free web-based randomizer [17]. There were no time constraints on the participants performance for the duration of the assessment. Appointments were scheduled 30 minutes apart.

### Statistical Analysis

Statistical analyses were undertaken using RStudio software (version 1.4; RStudio, PBC) [18] and tidyverse, blandr, ggplot2, and irr packages. Interrater reliability was determined between in-person (gold standard) and telehealth recorded results with a 1-way agreement intraclass correlation coefficient (ICC95), using the percentage method. If both raters recorded the same response, the ICC would be 100. For larger variations, the ICC would be lower. Each test had repetitions of the trials analyzed together, with missed trials excluded from the data. The ICCs were rated as excellent (>90), good (75-90), moderate (50-75), or poor (<50) [19]. Bland-Altman plots were created to assess agreement and biases between researchers. Intrarater reliability was assessed between consecutive telehealth trials for all measures in the same session. A sample size of 30 participants was chosen a priori for this reliability study, based on previous reliability trials using Functional Reach outcome measure (ie, 3 trials for intrarater data collection) [20].

## Results

### Participant Characteristics

A total of 30 individuals (11 female, 19 male) with a mean age of 36.2 (SD 12.5) years were recruited. Of them, 21 participants identified English as their first language, whereas 9 identified English as their second language, with various first languages including French, Mandarin, and Persian. Among the participants, 22 were familiar with at least one of the assessments prior to the study.

**Figure 1:**
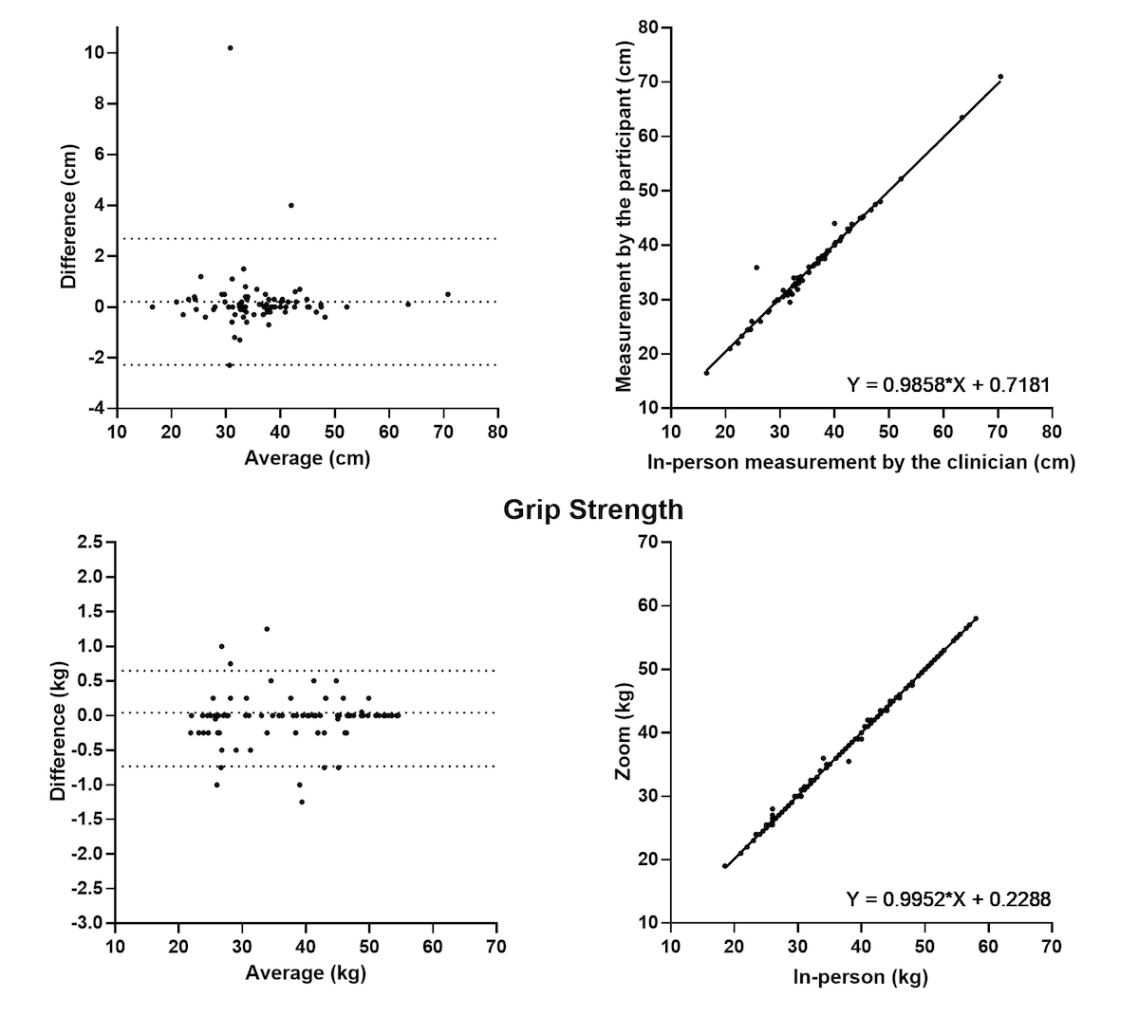
Interrater reliability and levels of agreement between in-person and Zoom measures for performance-based outcome measures.

**Figure 2:**
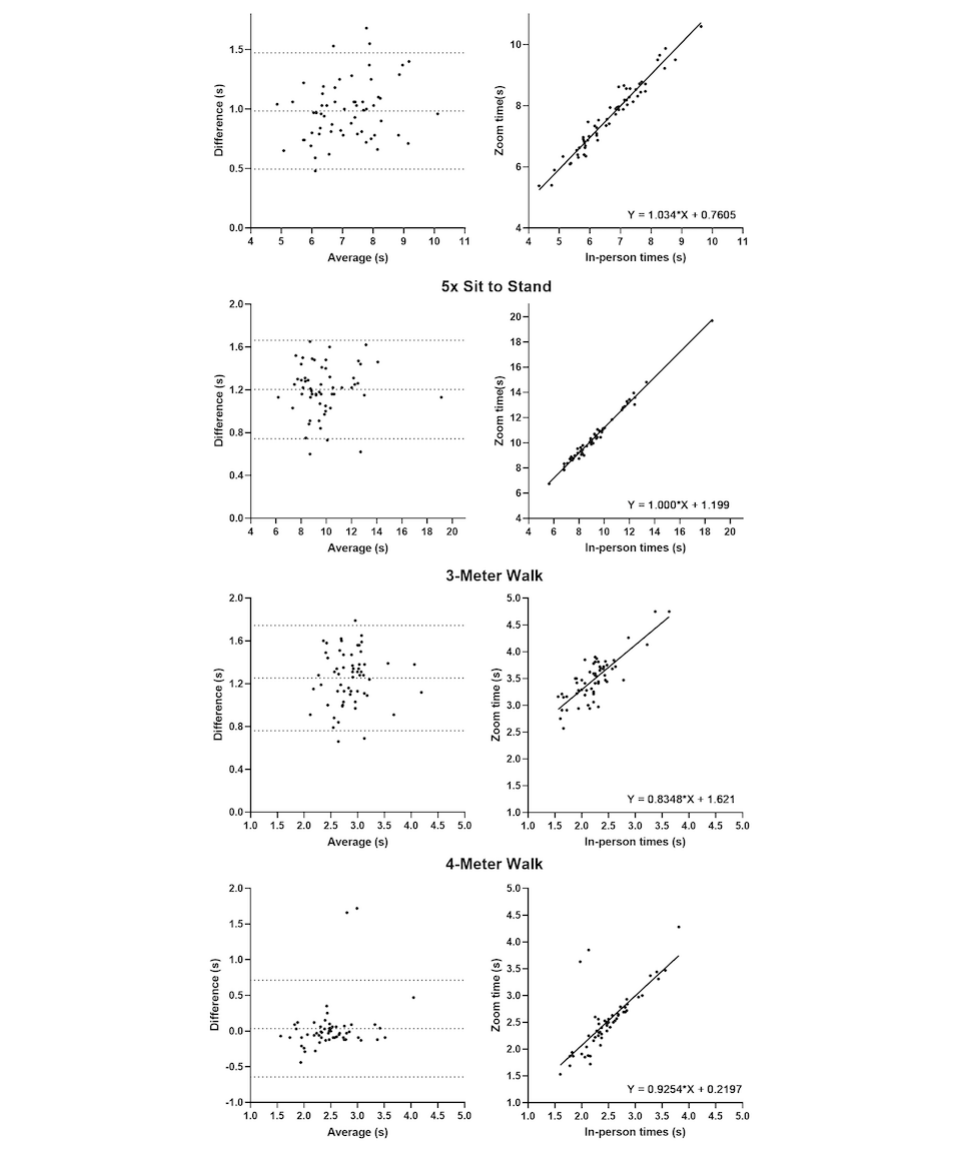
Interrater reliability and levels of agreement between in-person and Zoom measures for time-based outcome measures.

Excellent interrater reliability was seen for Grip Strength and Functional Reach Test (Table 2 and Figure 1). For the timed tests, there was good reliability in 5-Time Sit to Stand and 4-Meter Walk, moderate reliability in Timed Up and Go, and poor reliability in 3-Meter Walk (Table 2 and Figure 2). Intrarater reliability was excellent for Grip Strength; it was good for Timed Up and Go, 4-Meter Walk, and Functional Reach Test (ICC=0.84-0.89); and moderate for 5-Time Sit to Stand (Table 3). The number of trials for the intrarater reliability was determined by the use of standard protocols for face-to-face evaluation.

Bland-Altman analysis indicated a bias for the timed tests with a reaction time dependent component (ie, there was a lag between the Zoom instructor saying “go” and the participant moving). These biases led to longer times for the following telehealth results: 5-Time Sit to Stand (1.2 seconds), Timed Up and Go (1.0 seconds), and 3-Meter Walk (1.3 seconds).

A ceiling effect was observed for the static balance task from the Short Physical Performance Battery, and interrater and intrarater reliability could not be calculated.

**Table 2:** Intraclass correlation coefficient (ICC) for interrater (1:1) and intrarater reliability measures..

Measurements	Total trials, n	Telehealth measures, mean (SD)	In-person measures, mean (SD)	ICC^a^ (95% CI)	ICC^b^ (95% CI)
5-Time Sit to Stand (s)	58	10.46 (2.10)	9.25 (2.09)	0.84 (0.75-0.90)	0.67 (0.42-0.83)
Timed Up and Go (s)	60	7.61 (1.13)	6.63 (1.07)	0.64 (0.47-0.77)	0.84 (0.70-0.92)
3-Meter Walk Test (s)	59	3.48 (0.41)	2.23 (0.40)	–0.46 (–0.64-0.24)	0.89 (0.79-0.95)
4-Meter Walk Test (s)	58	3.48 (0.54)	2.48 (0.45)	0.76 (0.62-0.85)	0.86 (0.72-0.93)
Functional Reach Test (cm)	86	36.37 (8.22)	36.17 (8.24)	0.99 (0.98-0.99)	0.86 (0.76-0.92)
Grip Strength (kg)	180	38.47 (9.95)	38.43 (0.97)	0.99 (0.99-0.99)	0.91 (0.42-0.96)
Static Balance Test (points)	30	12 (0)	12 (0)	NA^c^	NA

^a^Interrater reliability of telehealth (Zoom) versus in-person trials.

^b^Intrarater reliability between telehealth trials.

^c^N/A: not applicable.

**Table 3:** Intrarater reliability of mobility-focused physical outcome measures recorded over telehealth technology (Zoom).

Assessment	Trial 1, mean (SD)	Trial 2, mean (SD)	Trial 3, mean (SD)
5-Time Sit to Stand (s)	10.82 (2.48)	9.92 (1.64)	N/A^a^
Timed Up and Go (s)	7.75 (1.23)	7.47 (1.02)	N/A
3-Meter Walk (s)	3.45 (0.42)	3.5 (0.40)	N/A
4-Meter Walk (s)	2.58 (0.58)	2.42 (0.50)	N/A
Functional Reach Test (cm)	35.58 (8.76)	36.6 3 (8.21)	37.44 (8.67)
Grip Strength (kg)	38.24 (9.92)	38.62 (10.20)	38.48 (9.90)
Static Balance Test (points)	12 (0)	N/A	N/A

^a^N/A: not applicable.

### Field Notes Describing Difficulties Encountered

The camera was in front of the participant for the walking tests and created some challenge for the remote evaluator due to depth perception issues. The remote evaluator recorded 3 instances of the camera freezing, which lasted less than 1 second. The angle of the laptop screen and integrated camera needed to be adjusted between some tests so that the appropriate body part could be seen. Instructions such as changing the chair orientation or distance from camera produced the correct adjustments, with instructions repeated only a couple of times by the remote evaluator. On 2 occasions, the in-person researcher, but not the telehealth researcher, noted that the participant lifted their heel during the Functional Reach Test. If the participant was further from the laptop, it was harder to hear their responses to questions. No adverse events such as pain or falls occurred during assessments. Two participants attended without their glasses and made small errors in reading the results from the Grip Strength dynamometer and the ruler on the Functional Reach Test.

## Discussion

### Principal Findings

Comparing telehealth to in-person assessments of strength, balance, and mobility resulted in reliability measures ranging from poor to excellent, depending on the type of assessment.

The results showed excellent reliability for Grip Strength and Functional Reach tasks; however, the reliability of mobility-focused physical outcomes assessed over telehealth, using Zoom, was poor to moderate compared to in-person assessments. Intrarater reliability for the Zoom assessments was moderate to excellent. For the Static Balance task, we could not conduct the interrater and intrarater reliability using the Bland-Altman analysis, due to a lack of data variability [21].

The interrater reliability was lower for measures that included a reaction time–dependent component, due in part to a time bias of longer times for telehealth recordings. Tests of a shorter duration were more impacted by the delay. Given the delay in some tests, caution is warranted when using traditional face-to-face assessments in a telehealth setting, and further information on the reliability and normative values of these assessments when delivered via telehealth is needed.

### Comparison With Prior Work

In this study, the assessments conducted via telehealth that were performance-based rather than time-based were extremely reliable and consistent. The highest reliability tests included the Grip Strength and Functional Reach Tests with excellent interrater reliability (ICC=0.99) in a telehealth setting, compared to in-person results. This finding is consistent with previous feasibility assessments and questionnaire-based reliability assessments [22] and is not surprising, given these assessments are not subject to any time delays over telehealth [23]. Practitioners can be extremely confident in the use of these assessments via telehealth for clinical practice.

Time-based mobility measures that were reaction time dependent had less reliable results. For example, in the Timed Up and Go task, the telehealth researcher started the timer when they said “go” and stopped the timer when the participant returned to their seat. These aspects of the task could be affected by network latency and could potentially increase and add variability to the time measured by the telehealth assessor, and it may explain the longer times consistently recorded by the remote evaluator. This finding adds to data from two small studies that found longer times for Timed Up and Go, albeit of smaller magnitudes (around 0.4 seconds), in a population of people after total knee replacement [7] and heart failure [9]. In combination, these studies suggest that it is not possible to directly compare data collected in person and via Zoom for the same individual, as these values are in the same order or magnitude as the minimally important clinical difference (eg, 0.6 of a second as calculated at 0.5 SD) [24]. These differences in values between in-person and Zoom data collection reduces the ability to use population norms from face-to-face data collection in making decisions related to data collection via telehealth. For clinical populations, where the overall time to complete these assessments is longer, the impact of the small lag will be less, resulting in higher reliability.

The stability of the lag reported by the remote evaluator and whether or not it is dependent on bandwidth or other features of the technology used remains unknown. Further research to quantify lag time in remote sessions compared with in-person testing is warranted if practitioners plan to use blended models of health service delivery (eg, a mix of face-to-face and telehealth) in the future. Alternatively, to avoid the time lag issues that we identified over videoconferencing, future research could investigate technologies that provide remote assessment without the need for timing over videoconferencing. For example, preliminary research is emerging regarding the use of mobile apps and body-worn sensor technology for walking and balance outcome measurement data in people who had a stroke [25].

### Future Directions

Telehealth assessments produced moderate to excellent intrarater reliability between trials. The Timed Up and Go and walking tests all produced good intrarater reliability between telehealth trials. Clinicians who provide services only via telehealth can be confident in the reliability of these tests when delivered via telehealth. The 5-Time Sit to Stand produced moderate intrarater reliability. This reduced reliability was likely due to a learning affect as participants’ second trial averaged 0.9 seconds quicker, which is consistent with previous reports [26]. Ensuring a practice trial is included before assessments would help reduce the learning effect between results in future research [27].

### Strengths and Limitations

This trial has collected data using robust methods; these methods may be appropriate to use when collecting data in clinical populations; however, the results of this study cannot be generalized to those cohorts. It is a limitation that the impact of changes in the internet connection or bandwidth on latency is unknown, potentially impacting the reproducibility of these results. Other potential sources of bias that may have influenced the results include the familiarity of some participants with some of the assessment items, contamination of intrarater’s second score, the difference in researchers’ experiences, and the fact that we could not test the reliability over a range of scores as is more likely in clinical practice.

### Recommendations

In this study, we identified considerations for practice to ensure high-quality and consistent telehealth assessments can be completed. The bias in some measures (eg, longer times of around 1 second via telehealth) has implications for blended practice and needs to be considered when comparing real changes in functions between in-person and remotely measured assessments. Measuring network latency prior to starting the assessment may be needed to help identify and correct for telehealth time biases. Measuring walking speed remotely remains challenging. Potential improvements include using a side-on view for walking tests to reducing the impact of depth perception issues. Further to this, it should always be ensured both parties can hear appropriately. External speakers or wireless headphones could assist in minimizing communication issues when the participant is at a distance from the computer. Lastly, the camera angle should be set in a way to show a participant’s full body, wherever possible.

### Conclusions

We provided important information on the reliability of mobility-focused physical outcome measures and recommendations of the utility of these measures for telehealth delivery. Practitioners can be very confident in undertaking performance-based measures based on our findings. Longer timed assessments produce the best reliability compared to shorter assessments. Consequently, practitioners should favor longer timed tests and protocols that do not depend on reaction times of the participant for the most optimal and consistent results. Further research is needed with clinical populations to assess reliability of the measures included in this study, with an appropriate balance assessment for the intended population. The biases detected in reaction time–dependent tests indicate that direct comparison with face-to-face delivery and comparison to normative data collected face-to-face cannot be made. High intrarater reliability of mobility-focused physical outcome measures have been demonstrated in this study.

## Abbreviations

ICC: intraclass correlation coefficient

## Data Availability

Data is available from corresponding author on reasonable request.
